# 

**Published:** 1893-12-02

**Authors:** 


**Notice to Advertisers and Subscribers.**

All Advertisements, Orders for Copies of The Hospital, and Business Communications should be addressed to The Publisher, *not to the Editor*, The Hospital, 428, Strand, W.C.

The Cost of Subscription, post free, is as follows:—Per week, 2½d.; per quarter, 2s. 6d.; per half-year, 5s.; per annum, 8s. 6d Foreign:—Per Annum, 10s. 8d.; payable, in all cases, in advance.

**OUR NEW OFFICES,**

428, Strand, W.C., will henceforth be the Office of this Journal.

**Notices of Births, Deaths, and Marriages** are inserted in this column at a charge of 2s. 6d. for an announcement not exceeding four lines.

BIRTH.

Brodie.—On November 25th, at 3, Chesterfield Street, Mayfair. W., the wife of Dr. Brodie, of a son.

DEATH.

Whitehouse.—On November 7th, at 48, John Street, Sunderland, aged 35 years, John Whitehouse, F.R.C.S.

### NOTICE TO CORRESPONDENTS.

All MS., Letters, Books for Review, and other matters intended for the Editor should be addressed The Editor, The Lodge, Porchester Square, London, W.

The Editor cannot undertake to return rejected MS., even when accompanied by stamped directed envelope.

**"Burdett's Hospital Annual," 1893.**

Fourth year of publication. 700 pp. Boards, gilt lettering. A most complete guide to British, Colonial, Indian, and American Hospitals, Dispensaries, Nursing and Convalescent Institutions, and Asylums. Price 5s. Published by The Scientific Press (Limited), 428, Strand, W.C.

**NOTICE.—The Index for Vol. XIV. (ending Sept. 30th, 1893) is on sale at the Office of this Journal, price 2½d., post free.**

### SCRAPS AND GLEANINGS.

A ball in aid of the funds of the Royal Hospital was held last month in the Town Hall at Portsmouth.

It is proposed shortly to hold another ball at Darlington in the interests of the Darlington Hospital.

We are glad to learn that a society for the prevention of cruelty to children has recently been founded in Liege.

The number of sanitary districts existing at the present time in England and Wales is 1,602, of which 573 are rural and 1,029 urban.

England and Wales are declared free from cholera, no case having been reported during the last fortnight, The extraordinary precautions will now be withdrawn.

A widespread epidemic of influenza prevails at Blackburn. The Medical Officer of Health states that it is more general than two years ago, but the disease is of a milder type.

A special meeting of the Halstead Local Board was held this month to take into further consideration the question of providing additional accommodation for the treatment of infectious diseases cases.

It is reported that the debt of £200 on the new wing of the Hawick Cottage Hospital and Dispensary has been reduced to £50 by special donations, including one of £50 from Mr. Glenny, of Buckland.

The sale of the patients' work, for their own benefit, will be held at the Institution in Clapham Road, on December 7th and 8th, and will be opened by Mrs. F. A. Bevan on the 7th, at half-past two o'clock.

The committee of the National Orthopædic Hospital are making an earnest appeal for increased financial support to clear the institution from the debt, amounting to £2,000, which exists on the accounts.

During the last year 66 patients have received treatment at the Faversham Cottage Hospital. The committee of this institute regret that the expenditure shows an increase over that of last year to the extent of £57.

At a meeting of the directors of Newport Infirmary, Isle of Wight, the secretary reported that Alderman T. Goldsworthy, one of the directors, had promised to endow a cot in the children's ward in celebration of his silver wedding.

The continual appeals solicited for financial support to enable the committee of the Bootle Borough Hospital to carry on their useful work shows that much money is required to comply with the calls upon the resources of the institution.

The medical profession in France will benefit largely under the will of Madame Buisson, recently deceased. This lady, the widow of the late Doyen of the Faculty of Medicine, has bequeathed £60,000 to establish and maintain a scientific and charitable foundation.

Lady Winchelsea is now engaged in conducting several conferences in Lincolnshire, and in furthering the prospects of the Rural Nursing Association of that county. The County Council has made a grant of £200 for the training of several nurses to operate in local service.

The committee of the Ballymena Cottage Hospital report that the financial position of the institution is better than it has been, though they regret a slight decrease in the annual subscriptions. The statement of accounts at the last annual meeting shows a balance in hand of £84.

During the past year the number of patients who have undergone treatment in the wards of the National Orthopædic Hospital, Great Portland Street, is 216, in addition to which 1,079 out-patients have received professional advice. The hospital is in urgent need of increased funds.

Great success attended the monster cyclist procession and lantern carnival which took place during the second week of November in aid of the funds of the Cardiff Infirmary. Donations were further solicited from the spectators, and we understand a substantial sum has been realised.

An adjourned conference of the Leigh Sanitary Authority and representatives of the Leigh, Atherton, and Tyldesley Local Board was held recently to consider the question of a joint infectious hospital for the district. The Leigh Sanatorium was offered for purchase in view of this erection being decided on.

The Sanitary Committee of the Barry and Cadoxton Local Board have determined to recommend that authority to schedule Sully Island for its compulsory purchase, it being proposed to erect thereon an infectious diseases hospital and a centre for isolating cholera cases. This island is not considered a suitable site for the proposed hospital.

Lieut.-General Sir Francis Norman, K.C.B., has been elected a member of the board of management of the British Home for Incurables, which charity has suffered a great loss by the recent death of Mr. T. H. Devonshire, who had long been a member of the board, and had taken a keen interest in the welfare of the Home from its foundation.

### Books Received.

Macmillan and Co.

"Adventures in Mashonaland." By two Hospital Nurses, Rose Blennerhassett and Lucy Sleeman. 8s. 6d. net.

W. and A. K. Johnston.

"Selected Papers in Gynæcology and Obstetrics." By D. Berry Hart M.D., F.R.C.P.Edin., &c.

Simpkin, Marshall, Hamilton, Kent, and Co.

"On Gout as a Peripheral Neurosis." By Willoughby Francis Wade.

Digby, Long, and Co.

"Dr. Janet of Harley Street." By Arabella Kenealy. Fifth Edition.

Scientific Press.

"Surgical Ward Work and Nursing." By Alexander Miles, M.D.

T. Fisher Unwin.

"Household Nursing." By John Ogle Tunstall, M.D.

Sampson Low and Marston.

"Camp Fires of a Naturalist." By C. E. Edwards.

J. and A. Churchill.

"Manual of the Diseases Peculiar to Women." By James Oliver, M.D.

Bailliere, Tindall, and Cox.

"Aids to the Diagnosis and Treatment of Diseases of Children. By John McCaw, M.D.

"Aids to Otology." By W. R. Stewart, F.R.C.S.E.

Charles Griffin and Co.

"The Hygienic Prevention of Consumption." By J. E. Squire, M.D.

Swann Sonnenschein.

"Suicide and Insanity." By S. Strahan, M.D.

W. Allen and Co.

"Sir Morell Mackenzie." By the Rev. H. R. Haweis, M.A. Second Edition.

**Periodicals and Pamphlets.**—Tit Bits, The Million, The Picture Magazine, The Strand Magazine, The Sun, Amateur Gardening, The Charity Organisation Review, The Clinical Journal, The Birmingham Medical Review, Cremation, by Sir Douglas Galton, The Religious Review of Reviews, The Health Record, Guy's Hospital Gazette, The Leisure Hour, The Sunday at Home, Girl's Own Paper (Christmas number), Boy's Own Paper (Christmas number), Klinischer Vorträge, Invention, Knowledge, Nature, The Gentlewoman (Christmas number), The Therapist, The Review of the Churches, and A New Method of Direct Fixation of the Fragments in Compound and Un-united Fractures, by N. Senn, M.D., Chicago.

### MEDICAL VACANCIES AND APPOINTMENTS.

### APPOINTMENTS.

T. D. Acland, M.A., M.D.Oxon., F.R.C.P.Lond., appointed Physician to St. Thomas's Hospital. Rosa Bale, L.R.C.P. and S.Edin., L.F.P. and S. Glas., appointed Senior House Surgeon to the Clapham Maternity Hospital. H. M. Body, M.R.C.S.Eng., L.S.A., re-appointed Medical Officer of Health for Crediton. J. Brown, M.D., D.S.Sci.Vict., re-appointed Medical Officer of Health for the Bacup Town Council, and Physician to the South ill Fever Hospital. F. W. Clark, M.D.Durh., appointed Medical Officer of Health for the Lowestoft Urban Sanitary District of the Mutford and Lothingland Union. J. A. Crump, L.R.C.P.Lond., M.R.C.S., appointed Assistant Medical Officer to the Wye House Asylum, Buxton. Mr. F. Fielder, appointed Resident Surgeon and Physician to the Brixton Dispensary. H. T. Hinton, M.B., C.M.Aber., appointed Medical Officer and Public Vaccinator for the Heytesbury District of the Warminster Union. Mr. J. H. Hunter, M.B., B.S.Dunelm, of Newcastle-upon-Tyne, appointed Junior House Surgeon at the Ingham Infirmary. W. Kirkpatrick, M.D., appointed Honorary Surgeon to the Stourbridge Dispensary. J. H. Lightbody, M.D.Vict., M.R.C.S., L.R.C.P., L.S.A., appointed Medical Officer and Public Vaccinator to the Wybunbury District of the Nantwich Union. Dr. Little, appointed Medical Officer of Health for Gordon. R. Cadell Macdonald, M.B., C.M., L.R.C.S.E., L.R.C.P.E., appointed Parochial Medical Officer for the Parish of Northmavine, Shetland. W. C. McDonnell, L.R.C.P., M.R.C.S., appointed Medical Officer for the No. 6 District of the Hackney Union. Mr. A. H. Minton, appointed Medical Officer for the South District of the Stratton Union. J. S. Morrow, B.A., M.B., B.Ch., appointed Honorary Assistant Surgeon to the Belfast Hospital for Sick Children. B. Pitts, M.A., M.B.Cantab., F.R.C.S.Eng., appointed Surgeon to St. Thomas's Hospital. H. A. Robinson, M.B., Ch.B.Vict., appointed House Surgeon to the Chorley Hospital and Dispensary. Mr. A. E. Shepherd, appointed Junior Assistant Medical Officer at the Infirmary of the Fulham Union. Dr. L. D. Temple, appointed Medical Officer of Health for Saddell. H. Tilley, M.D., B.S.Lond., M.R.C.S., L.R.C.P.Lond., appointed Junior Assistant Surgeon to the London Throat Hospital, Great Portland Street. G. Vincent, M.D., M.R.C.S., D.P.H.Camb., appointed Parochial Medical Officer to the Bramford and Flowton District of the Bosmere and Claydon Union. J. W. Watterson, M.B., C.M.Edin., appointed Medical Officer of Health to the Morecambe Local Board. W. H. Webb, L.R.C.P.Lond., M.R.C.S.-Eng., appointed Medical Officer of Health to the Kingsbridge Local Board. Winifred Westlake, L.R.C.P. and S.Edin., L.F.P. and S.Glasg., appointed Junior House Surgeon to the Clapham Maternity Hospital.

### VACANCIES.

#### Resident Medical Officers.

Albany General Hospital, Grahamstown, South Africa. Resident Medical Officer. Salary £200 per annum, increasing yearly by £50 up to £300, with furnished apartments. The selected candidate will have a free first-class passage. Applications to Dr. Macphail, Rowditch, Derby.

Birmingham and Midland Free Hospital for Sick Children, Steelhouse Lane, Birmingham. Resident Medical Officer and Resident Surgical Officer. Salaries £70 and £50 respectively, with board, washing, and attendance. Applications to the Secretary by December 6th.

Chelsea Hospital for Women, Fulham Road, S.W. Resident Medical Officer. Salary £80 per annum, with board and residence. Appointment for one year. Applications to the Secretary by December 5th.

Chester General Infirmary. House Surgeon; doubly qualified. Salary £90 per annum, advancing £10 yearly, with residence and maintenance in the house. A sum of £5 per annum is granted for taking charge of the library. Applications to the Chairman of the Board of Management by December 4th.

City of London Hospital for Diseases of the Chest, Victoria Park, E. House Physicians. Board and residence, and allowance for washing provided. Appointment for six months. Applications to the Secretary at the Office, 24, Finsbury Circus, E.C., by December 14th.

Glasgow Eye Infirmary. Resident House Surgeon. Salary £50 per annum, with apartments and board. Applications to W. G. Black, Secretary, 88, West Regent Street, Glasgow, by December 9th.

Kimberley Hospital, Kimberley. Senior House Surgeon. Salary £650 per annum, with unfurnished quarters for single men. Applications to H. A. De Beer, Secretary, by December 25th.

National Sanatorium for Consumption and Diseases of the Chest, Bournemouth. Resident Medical Officer and Secretary. Salary £120 per annum, with board and lodging. Applications to the "Chairman of Committee" by December 5th.

Royal Free Hospital, Gray's Inn Road, W.C. Senior Resident Medical Officer; doubly qualified. Salary £100 per annum, with board, residence, and washing. Appointment for twelve months, but eligible for re-election. Applications to the Secretary by December 11th.

Sunderland Infirmary. House Physician; doubly qualified. Salary £80 per annum, rising £10 annually to £100, with board and residence. Applications to the Chairman of the Medical Board by December 4th.

#### Non-Resident Medical Officer.

Birmingham and Midland Free Hospital for Sick Children, Steelhouse Lane, Birmingham. Extra Acting Physician. Salary, £60. Applications to the Medical Committee by December 6th.

### PASS LISTS.

### University of London.

INTERMEDIATE EXAMINATION IN SCIENCE, 1893.

First Division.—J. H. Ashworth, Owens College; Gertrude Attwater, University College; F. Barlow, Herne House, Margate, and University Tutorial College; N. Baron, University Tutorial College and private study; W. Bradley, University Tutorial College and Goldsmiths' Institute; G. B. Bryan, University College, Nottingham; Mabel R. Byles, University College, Aberystwith; J. W. Caithness, B.A., private study and tuition; A. L. Cann, B.A., St. John's College, Battersea, and University Tutorial College; A. W. Carr, Brighton Grammar School; C. W. Clarke, Mason College and Birmingham Technical School; E. L. Cotton, Firth College and private study; J. M. Crofts, Sir T. Rich's School, Gloucester, and University Tutorial College; E. Crosland, Birkbeck Institute and private study; H. M. Dawson, Yorkshire College; E. M. Eagles, St. John's College, Cambridge; H. L. Eason, University College; E. A. Eden, B.A., University College and private study; W. Erskine, private study; T. J. Evans, University College, Aberystwith; Emily C. Fortey, University College, Bristol, and private study; M. B. Fowler, University Tutorial College and Goldsmiths' Institute; O. Gledhill, Yorkshire College and private study; G. Grace, Royal College of Science and Yorkshire College; J. G. Greenhalgh, Clare College, Cambridge, and University Tutorial College; Lucy Hall, Sheffield High School and Firth College; E. F. Harrison, Pharmaceutical Society, Birkbeck Institute, and private study; H. E. Hewitt, St. Thomas's Hospital and privaie study; F. A. Hillard, B.A., Merton College, Oxford; F. A. Hocking, Pharmaceutical Society, King's College, and private tuition; A. Howell, St. Mary's Hospital and University Tutorial College; H. S. Jevons, University College; Amy F. M. Johnson, Ladies' College, Cheltenham; S. Jones, University Colleges, Cardiff and Aberystwith; Alice F. Joseph, Bedford College, London; Charles E. Kemp, University College, Aberystwith; W. Lefèvre, Yorkshire College and Huddersfield Technical School; A. J. Linford, University Tutorial College and private study; G. McFarlane, Owen's School, Birkbeck Institute, and private study; H. Main, Birkbeck Institute, University Tutorial College, and private study; C. Mills, Central and Birkbeck Institutes and private study; S. R. Milner, University College, Bristol; J. W. Nuttall, private study; G. H. Perry, Royal College of Science; A. Philip, Royal School of Mines and Central Institute; T. S. Price, Mason College; Adelaide J. Raisin, private study; W. B. Randles, University College, Aberystwith; Amy C. Robbins, Middlesex College of Chemistry, University Tutorial College, and private tuition; J. H. Robbins, private study and University Tutorial College; C. Rodgers, Firth College; Fabian Rosenberg, Emanuel College, Cambridge; J. S. Ross, Glasgow Technical College and private study; Gertrude H Rowland, Royal Holloway College; C. J. Seargent, private study; E. A. Smith, University College, Nottingham; A. Stansfield, private study and University Tutorial College; E. F. Stubbs, University Tutorial College and private study; A. K. B. R. W. Taylor, private study and University Tutorial College; W. P. Welpton, Yorkshire College; G. H. West, Mason College; J. Williams, St. John's College, Battersea, and University Tutorial College; A. G. Woodhead, Owens College; T. W. Wormell, private study and University College, Nottingham.

Second Division.—B. Amsden, LL.B., B.A., private study; W. Baird, private study, and Anderson's College and Grove Street Public School, Glasgow; E. C. P. Barber, Yorkshire College and private study; T. Barrs, B.A., private study; J. I. Bates, Mason College and private study; J. B. Baylis, Firth College and private study; F. A. Beesley, University College, Nottingham; H. W. Boddy, St. Mark's College, Chelsea, and University Tutorial College; J. O. Borley, private tuition and University Tutorial College; J. H. Brittain, Harris Institute, Preston, and private study; Florence M. Clark, University College; A. Clough, Yorkshire College, Bradford, and Keighley Technical Schools and private study; A. Coleman, University Tutorial and City of London Colleges; F. Collingridge, University College; G. T. Cooper. private study and tuition; E. H. Davies, University College, Cardiff; G. R. Davies, Mason College; N. Davies, University College, Cardiff; Ethel M. Ecroyd, Sheffield High School and Firth and Westford Colleges; S. R. Edminson, University Tutorial and Firth Colleges, and University College, Liverpool; D. Ellis, University College, Aberystwith; R. Evans, private tuition and study, and Stockport Technical School; J. A. Foster, University College; Katie E. R. Garaway, University College, Bristol; A. E. Garrett, Borough Road and Firth Colleges and private study; C. A. Ginever, private study; W. Goddard, Middlesbrough Science School; W. C. Griffiths, private study; G. E. Grimes, Royal College of Science and Birkbeck Institute; H. H. Higgs, B.A., Wyggeston School and private study; A. E. Johnson, private study; W. Johnson, B.A., private study and Royal College of Science; T. G. Joyce, Mason College; T. Judge, non-collegiate, Oxford; C. Kaye, St. John's College, Battersea, and private study; Rosalie B. J. Lulham, Royal Holloway College; H. K. Marsden, Owens College; W. Mitchell, private study; Margaret E. Moore, private study and University College, Liverpool; G. Murdoch, B.A., Andersonian University and private study; S. T. Parkinson, University College, Aberystwith; Gertrude E. Ready, Royal Holloway College; Mary F. Saunders, University Tutorial College and Gloucester Public School of Science; A. H. Scholefield, B.A., private study and University Tutorial College; F. H. Shoosmith, private study; A. Smith, Clifton Laboratory, Merchant Venturers' School, and private study; A. Stead, Yorkshire College; A. H. Summers, University College, Aberystwith, and private study; R. Walmsley, Owens College; Beatrice A. Ward, Ladies' College, Cheltenham.

Honours Candidates Recommended for a Pass.—A. E. Brown, private study; A. T. Quelch, University College and City of London School; A. Salanson, Merchant Venturers' School, Bristol, and private tuition; G. D. Thomas, Merchant Venturers' School, Bristol; J. L. Holt, Yorkshire College.

